# Detection of Rat Lungworms in Invasive Mollusks, Georgia, USA, 2024

**DOI:** 10.3201/eid3109.250133

**Published:** 2025-09

**Authors:** Tyler J. Achatz, Caley H. Chun, Maggie A. Young, Jim Page, Matthew Rowe, Caroline Cooper, Laura Wenk, Vasyl V. Tkach

**Affiliations:** Middle Georgia State University, Macon, Georgia, USA (T.J. Achatz, C.H. Chun, M.A. Young); Georgia Department of Natural Resources, Atlanta, Georgia, USA (J. Page, M. Rowe, C. Cooper, L. Wenk); University of North Dakota, Grand Forks, North Dakota, USA (V.V. Tkach)

**Keywords:** Angiostrongylus cantonensis, rat lungworm, parasites, zoonoses, meningitis/encephalitis, mollusks, Mollusca, lakes, rivers, DNA, prevalence, surveillance, larvae, Georgia, United States

## Abstract

The rat lungworm, *Angiostrongylus cantonensis*, is an invasive, zoonotic parasite that can cause severe disease in humans. We collected *A. cantonensis* larvae from 2 host species, invasive apple and mystery snails, from bodies of water in Georgia, USA. Recreational water users should avoid ingesting potentially infected hosts, aquatic vegetation, and water.

The rat lungworm, *Angiostrongylus cantonensis* (Nematoda: Angiostrongylidae), is an invasive human pathogen in many countries, including the United States. This nematode naturally parasitizes rodents (*1*–*3*); a variety of gastropod mollusks, typically terrestrial gastropods, act as intermediate hosts. However, aquatic and semiaquatic mollusks, such as invasive apple snails (*Pomacea* spp.) and mystery snails (*Cipangopaludina* spp.), have been reported as intermediate hosts (*4*,*5*). Freshwater crustaceans, amphibians, reptiles, and flatworms might serve as paratenic hosts (*1*–*4*). Infective third-stage level (L3) nematode larvae can also be found on vegetation exposed to infected snails (*1*). When L3 larvae are ingested by rats, the larvae migrate through vasculature, reaching the central nervous system, and later develop into adults in the pulmonary arteries. In humans, accidental ingestion of rat lungworm can cause severe pathology, including meningitis, or death when L3 larvae migrate to the central nervous system (*6*).

Rat lungworms are native to Southeast Asia but have spread worldwide (*4*); the parasite was first reported in the United States in Hawaii in 1960 (*2*). It was not detected again until 1986 in Louisiana. Recent years have seen a geographic expansion of this parasite: 2013 in Florida, Mississippi, and Texas; 2014 in Alabama and California; 2015 in Oklahoma; 2019 in South Carolina; and 2019–2022 in Georgia (*2*,*4*,*5*,*7*–*9*). Despite the broad geographic distribution of rat lungworm, few cases of human angiostrongyliasis have been detected in the United States (*8*). We collected 2 rat lungworm host species, invasive apple snails (*Pomacea maculata*) and mystery snails (*Cipangopaludina japonica*), in bodies of water in Georgia and tested them for *A. cantonensis* larvae.

We collected the snails from 8 water bodies in 7 counties during May–October 2024 (Table; Figure). We sampled 430 apple snails (Camden, Chatham, and Dougherty Counties) and 2,562 mystery snails (Cherokee, Greene, Hall, and Jasper Counties) and screened them for nematodes (Appendix, https://wwwnc.cdc.gov/EID/article/31/9/25-0133-App1.pdf). A total of 14 snails (5 mystery snails, 9 apple snails) were infected with rat lungworm. No variation was detected among *cox*1 sequences from the nematodes. BLAST analysis (https://blast.ncbi.nlm.nih.gov) showed a 100% match to *A. cantonensis* parasites previously collected in Atlanta (*9*). Among sites sampled for mystery snails, we detected rat lungworm from Lake Lanier (Hall County; prevalence 18.0/1,000 snails) and the Ocmulgee River (Jasper County; prevalence 6.3/1,000 snails), whereas mystery snails from Lakes Allatoona (Cherokee County) and Oconee (Greene County) were not infected. Apple snails taken from ponds and marshes in Kingsland (prevalence 189.2/1,000 snails) and St. Marys (prevalence 8.5/1,000 snails), both in Camden County, and from Pipemakers Canal (Chatham County; prevalence 4.5/1,000 snails), were infected with rat lungworm, but those from Lake Chehaw (Dougherty County) were not infected (Table). Despite our broad sampling of snails, we detected low overall prevalence of rat lungworm.

**Table:** Prevalence of rat lungworm (*Angiostrongylus cantonensis*) in invasive apple snails (*Pomacea maculata*) and mystery snails (*Cipangopaludina japonica*), Georgia, USA*

Location	County	GPS coordinates	Snail type	No. infected/no. screened	Prevalence, infections/1,000 snails
Lake Allatoona	Cherokee	34°07′58.1′′N, 84°37′46.5′′W	Mystery	0/1,371	0
Lake Lanier	Hall	34°17′35.0′′N, 83°56′17.6′′W	Mystery	2/111	18
Lake Oconee	Greene	33°30′16.5′′N, 83°16′58.0′′W	Mystery	0/607	0
Ocmulgee River	Jasper	33°19′07.4′′N, 83°50′32.6′′W	Mystery	3/473	6.3
Kingsland	Camden	30°47′17.0′′N, 81°38′46.0′′W	Apple	7/37	189.2
Lake Chehaw	Dougherty	31°36′37.2′′N, 84°06′56.3′′W	Apple	0/55	0
Pipemakers Canal	Chatham	32°06′21.0′′N, 81°11′43.0′′W	Apple	1/221	4.5
St. Marys	Camden	30°47′07′′N, 81°35′25.0′′W	Apple	1/117	8.5

*GPS, global positioning satellite.

**Figure:**
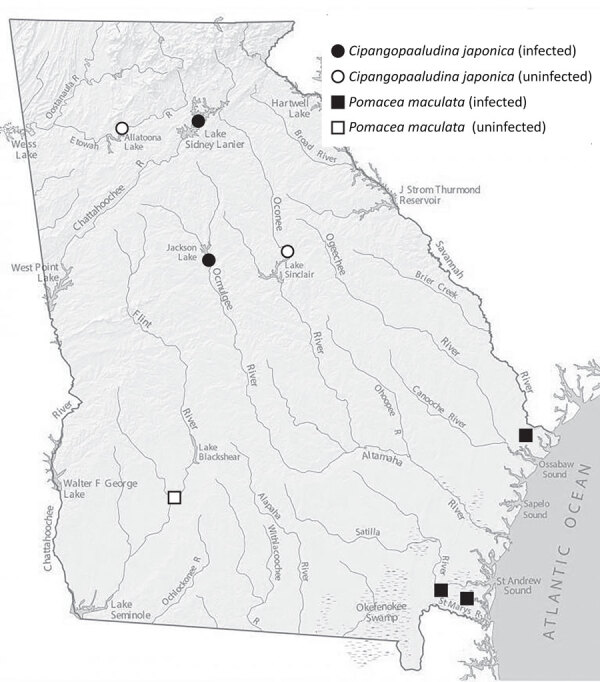
Sites where invasive apple snails (*Pomacea maculata*) and mystery snails (*Cipangopaludina japonica*) were collected and screened for rat lungworm (*Angiostrongylus cantonensis*), Georgia, USA.

Apple snails were first reported in Georgia in 1974 but not reported again until 2005 (*10*). The early records of the snails were limited to southern Georgia, but in 2013, apple snails were reported in Rockdale County in north central Georgia (*10*). In the early 2020s, apple snails began to be reported more frequently in the central and northern parts of the state, including in and around the Atlanta metropolitan area (*10*). Mystery snails are a more recent introduction to Georgia; they were first reported in the state in 2013 from the Atlanta metropolitan area (Clayton and Fulton Counties) (*10*). Reports of mystery snails throughout the state have become increasingly frequent in recent years (*10*). We anticipate that future sampling of these snails statewide will show a general trend of increasing prevalence as the invasive snail populations become more established and widespread.

Humans can be infected with rat lungworm by ingesting the molluscan intermediate host or the paratenic host (e.g., crustaceans) or by swallowing infective (L3) larvae, which are found on vegetation (*1*–*3*). Apple snails are commonly consumed in some communities in the United States, including Georgia (C.H. Chun, J. Page, M. Rowe, pers. observ.). However, risk of contracting rat lungworm infection is low. Thoroughly cooking the infected snail or paratenic host kills the nematodes and prevents infection; however, accidental exposure through ingesting contaminated vegetation poses a greater human health risk (*1*). Prior studies have suggested potential human infection through contaminated drinking water (*3*). 

In conclusion, whereas public health decisions related to this parasite should be left to the Centers for Disease Control and Prevention and the Georgia Department of Public Health, we encourage efforts to educate recreational water users to avoid ingesting potentially infected hosts, aquatic vegetation, and water. Long-term management and monitoring of the invasive snail and rodent populations are needed to help minimize the potential spread of rat lungworm and human infection risk in Georgia.

Appendix Additional information for detection of rat lungworms in invasive mollusks, Georgia, USA, 2024.
