# Supplementary material for: Detection of Rat Lungworms in Invasive Mollusks, Georgia, USA, 2024
Source: Emerg Infect Dis. 2025 Sep;31(9):1852–4. doi: 10.3201/eid3109.250133 (PMC12407195; doi:10.3201/eid3109.250133)
Supplement: Appendix — Additional information for detection of rat lungworms in invasive mollusks, Georgia, USA, 2024. [file 25-0133-Techapp-s1.pdf]

Article DOI: https://doi.org/10.3201/eid3109.250133

EID cannot ensure accessibility for supplementary materials supplied by authors. Readers who have difficulty accessing supplementary content should contact the authors for assistance.

Detection of Rat Lungworms in Invasive Mollusks, Georgia, USA, 2024

Appendix

Collection Methods

Apple snails were collected at night after emerging from water to lay eggs, while mystery snails were collected in the water. Snails were briefly rinsed in water and individually placed into containers with saline. After at least 4 hours, the containers were visually examined for presence of free-swimming larval stages with use of a Stemi 305 Stereo Zoom Microscope (8–40x total magnification) (Zeiss, Baden-Württemberg, Germany). Snails were manually crushed and inspected for internal nematodes. Nematodes were placed directly into 80% ethanol and stored at −20°C for molecular analysis.

Molecular Methods

Genomic DNA was extracted from individual nematodes using an ethanol precipitation method. At least one specimen from each snail infected with nematodes was extracted. A fragment of the cytochrome *c* oxidase subunit 1 (*cox*1) mtDNA gene was amplified by polymerase chain reactions (PCR) in a T100 thermal cycler (Bio-Rad, California, USA) using forward primer CO1ACF7 (5′-TGCCTGCTTTTGGGATTGTTAGAC-3′) and reverse primer CO1ACR7 (5′-TCACTCCCGTAGGAACCGCA-3′) (*1*). PCRs were performed in a total volume of 25 µl using GoTaq G2 DNA Polymerase (Promega, Wisconsin) according to the manufacturer’s instructions and an annealing temperature of 58°C.

PCR products were purified using an Illustra ExoProStar PCR clean-up enzymatic kit (Cytiva, Massachusetts); subsequently a BrightDye® terminator chemistry kit (Molecular Cloning Laboratories, California) was used to cycle-sequence the purified PCR products. PCR

primers were used for sequencing reactions. The sequencing reactions were purified with a MCLab BigDye® sequencing clean up kit and run on an ABI 3130 automated capillary sequencer (Thermo Fisher Scientific, Massachusetts). Contiguous sequences were assembled using Sequencher ver. 4.2 (GeneCodes Corp., Michigan) and deposited in GenBank (accessions PQ851676–PQ851681).

References

1. Gottdenker NL, Nascimento Ramos RA, Hakimi H, McHale B, Rivera S, Miller BM, et al. *Angiostrongylus cantonensis* infection in brown rats (*Rattus norvegicus*), Atlanta, Georgia, USA, 2019–2022. Emerg Infect Dis. 2023;29:2167–70. 10.3201/eid2910.230706 PubMed
